# A Manual of Elementary Chemistry

**Published:** 1859-09

**Authors:** 


ART. V.—*A Manual of Elementary Chemistry, Theoretical and Practical.* By GEORGE FOWNES, F.R.S., etc. Edited by ROBERT BRIDGES, M.D., etc. 8vo., pp. 600. Philadelphia: Blanchard & Lea. 1859.

THE present edition of this popular manual is a reprint from the seventh London edition, which, having been carefully revised by its English editors, Drs. Jones and Hoffman, will be found to be complete as regards the necessary additions and alterations that the progress of chemical science in the past few years demands. As a practical outline of chemistry and a faithful guide to the student, it is too well known to require commendation on our part. Being recommended as a text-book in many of our institutions, it has become a familiar companion to many who will eagerly seek the new edition, which has been carefully supervised in its passage through the press by that accomplished and well-qualified chemist, Dr. Bridges.

